# Diversity of Phospholipases A_2_ from *Bothrops atrox* Snake Venom: Adaptive Advantages for Snakes Compromising Treatments for Snakebite Patients

**DOI:** 10.3390/toxins14080543

**Published:** 2022-08-08

**Authors:** Leijiane F. Sousa, Amanda P. Freitas, Bruna L. Cardoso, Tiago H. M. Del-Rei, Vanessa A. Mendes, Daniele P. Oréfice, Marisa M. T. Rocha, Benedito C. Prezoto, Ana M. Moura-da-Silva

**Affiliations:** 1 Laboratório de Imunopatologia, Instituto Butantan, Av. Vital Brazil, 1500, São Paulo 05503-900, SP, Brazil; 2 Laboratório de Dor e Sinalização, Instituto Butantan, Av. Vital Brazil, 1500, São Paulo 05503-900, SP, Brazil; 3 Laboratório de Herpetologia, Instituto Butantan, Av. Vital Brazil, 1500, São Paulo 05503-900, SP, Brazil; 4 Laboratório de Farmacologia, Instituto Butantan, Av. Vital Brazil, 1500, São Paulo 05503-900, SP, Brazil

**Keywords:** PLA_2_, diversity, snakebites, snake venom, antivenom

## Abstract

The evolution of snake venoms resulted in multigene toxin families that code for structurally similar isoforms eventually harboring distinct functions. PLA_2_s are dominant toxins in viper venoms, and little is known about the impact of their diversity on human envenomings and neutralization by antivenoms. Here, we show the isolation of three distinct PLA_2_s from *B. atrox* venom. FA1 is a Lys-49 homologue, and FA3 and FA4 are catalytic Asp-49 PLA_2_s. FA1 and FA3 are basic myotoxic proteins, while FA4 is an acid non-myotoxic PLA_2_. FA3 was the most potent toxin, inducing higher levels of edema, inflammatory nociception, indirect hemolysis, and anticoagulant activity on human, rat, and chicken plasmas. FA4 presented lower anticoagulant activity, and FA1 had only a slight effect on human and rat plasmas. PLA_2_s presented differential reactivities with antivenoms, with an emphasis on FA3, which was not recognized or neutralized by the antivenoms used in this study. Our findings reveal the functional and antigenic diversity among PLA_2_s from *B. atrox* venom, highlighting the importance of assessing venom variability for understanding human envenomations and treatment with antivenoms, particularly evident here as the antivenom fails to recognize FA3, the most active multifunctional toxin described.

## 1. Introduction

Snake venoms have evolved a wide diversity of proteins that function by disrupting homeostatic physiological processes to rapidly incapacitate prey [1]. In these venoms, the pharmacological and toxicological effects are caused by hundreds of components distributed in a few protein families, in which multiple protein isoforms can be represented in venoms [2]. The dominant families are secreted phospholipases A_2_ (PLA_2_), snake venom metalloproteinases (SVMPs), snake venom serine proteases (SVSPs), three-finger peptides (3FTXs), and C-type lectins (CTLs), while cysteine-rich secretory proteins, L-amino acid oxidases, Kunitz peptides, and others may also be present, but in lower abundances and with a reduced number of isoforms [3,4]. Under different conditions, the abundance of each isoform in the venoms is variable, thus corresponding to additional quantitative aspects of venom variability [2,5]. Snake venom composition varies interspecifically [6], as well as intraspecifically, with many factors influencing this diversity, including age [7], gender [8], location [9,10], diet [11], habitat [4], and season [12].

The diversity in venom composition results in a multifunctional cocktail that induces harmful neurotoxic and hemotoxic effects, such as hemorrhage and coagulopathy, associated with severe pain, paralysis, myotoxic effects, inflammation, and necrosis. In human accidents with snakes, envenomings may result in death or severe morbidity, remaining a major public health problem, in which the available treatment is antivenom administration [13]. PLA_2_s are dominant toxins present in most snake venoms. They belong to the secreted group of PLA_2_s and are classified into two main subgroups: IA—from elapids, and IIA—found in viper venoms. Considering the evolutionary history of PLA_2_s, there is evidence that they have evolved from pancreatic enzymes recruited for the venom repertoire [1,3,6,14]. These genes underwent extensive duplication and successive mutations over time, generating the different isoforms, and eventually, neofunctionalization [15,16]. Most of the PLA_2_ isoforms are catalytically active with a conserved aspartic acid at position 49 (Asp-49), and particularly in vipers, some are catalytically less active, due to a substitution of the aspartic acid by lysine (Lys-49) [17]. In general, snake venom PLA_2_s are low molecular mass proteins, varying between 14 and 18 kDa [18]. However, dimeric forms, particularly common in Lys-49 isoforms, are not rare in *Bothrops* venoms [19,20].

The PLA_2_s induce diverse toxic and pharmacological effects. In the venoms of elapids, they are mostly pre-synaptic β-neurotoxins targeting the motor nerve terminals at the neuromuscular junction [21]. In vipers, they exert multiple effects such as neurotoxicity, cardiotoxicity, hemolysis, platelet aggregation inhibition, anticoagulation, edema, inflammation, and myotoxicity [22], and together with SVMPs, these toxins are largely responsible for the intense local pain and tissue damage caused by the venoms of *Bothrops* snakes [23]. In *Bothrops* snake venoms, most PLA_2_s exert myotoxic effects that may lead to necrosis and permanent tissue damage [24]. All Lys-49 PLA_2_s studied to date are basic proteins that induce muscle necrosis at the inoculation site, whereas only some Asp-49 are myotoxic basic proteins [17]. Lys-49 PLA_2_s induce myonecrosis via mechanisms independent of catalytic activity, involving the amino acids of the C-terminal region [25,26]. On the other hand, Asp-49 isoforms are partially dependent on their catalytic activity to disrupt the integrity of the muscle membrane, since the inhibition of catalytic activity may result in a decrease in residual myotoxicity [20,27].

At the systemic level, some PLA_2_s trigger the lysis of red blood cells by compromising the components and structure of the cell membrane, as well as altering ion flow and the intracellular metabolism [28,29]. In addition, the arachidonic acid resulting from the breakdown of phospholipids present in the cell membrane results in the release of arachidonic acid and triggers an inflammatory cascade of mediators acting on tissue damage and pain [30]. On platelets, the hydrolysis of membrane phospholipids may result in anticoagulant effects, as key steps of the blood coagulation cascade depend on negatively charged phospholipid membrane surfaces [28,31].

The features of snake venom PLA_2_s have been studied in a few venoms, and some isoforms have already been isolated and characterized from the *Bothrops atrox* venom [32,33,34,35,36]. However, their functional diversity and consequences on human envenomings and antivenom therapy are not yet fully understood. In a previous study [4], we compared the composition and functional activities of venoms from *B. atrox* snakes collected in four habitats at Pará State, Brazilian Amazon: forest, floodplain, pasture, and a degraded area. We observed in the whole venoms, important differences in their PLA_2_s catalytic activities and myotoxicities. Additionally, there was a lower reactivity of the commercial *Bothrops* antivenom with the chromatographic fractions in which PLA_2_s were eluted (Supplementary Figure S1). In this study, we aimed to investigate the extent of functional variability in *B. atrox* venom from different habitats, using isolated PLA_2_ isoforms. Our data bring new insights into the implications of PLA_2_s diversity as an adaptive advantage for the species, and the consequences for antivenom therapy the in cases of snakebites.

## 2. Results

### 2.1. Identification of Three Distinct PLA_2_ Isoforms in B. atrox Venom

**Figure 1:**
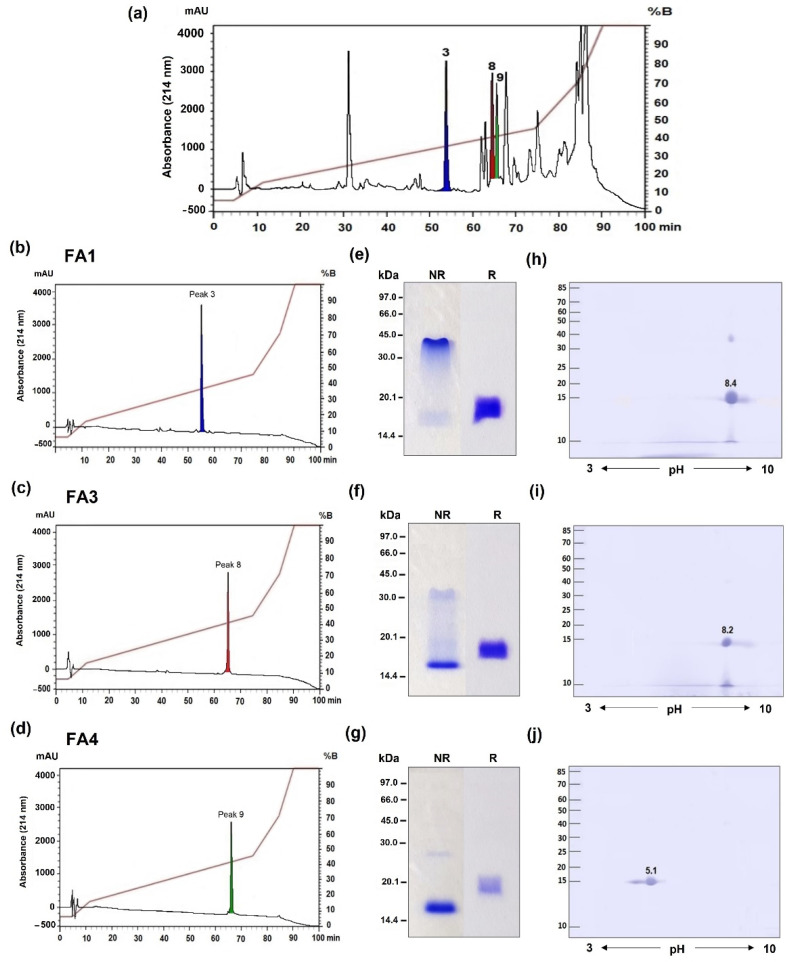
Chromatographic and electrophoretic profiles of the PLA_2_s isolated from *B. atrox* venom. *B. atrox* venom was submitted to RP-HPLC in a C18 column using a gradient elution of solution A (0.1% TFA) and solution B (100% acetonitrile) at a flow rate of 2.0 mL/min (**a**). Three fractions (peaks 3, 8, and 9) were collected, dried in a SpeedVac, resuspended in 1 mL of solution A, and then rechromatographed individually using the same protocol: peak 3 (FA1) eluted at 55.6 min (**b**); peak 8 (FA3) eluted at 65.8 min (**c**); and peak 9 (FA4) eluted at 67.49 min (**d**). Isolated toxins were subjected to 1D-SDS-PAGE in 15% polyacrylamide gels under reducing (R) and non-reducing (NR) conditions (FA1-**e**; FA3-**f**; FA4-**g**) and 2D-PAGE (FA1-**h**; FA3-**i**; FA4-**j**), with a pH gradient of 3–10 in the first dimension, and 15% polyacrylamide-SDS gels for the second dimension. Gels were stained with Coomassie Blue G250. The mobility of molecular weight (MW) standards is shown on the left, with values indicated in kDa.

*B. atrox* venom was fractionated via RP-HPLC using an RP100 C18 column, resulting in 20 major fractions accordingly to our previous study [4], in which, the peaks 3 (RT = 55 min), 8 (RT = 65 min), and 9 (RT = 67 min) contained the relevant PLA_2_s fractions previously identified, and they represent the PLA_2_s analyzed in this study (Figure 1a). The fractions were collected and resubmitted separately to the same chromatography, resulting in three isolated toxins, termed FA1 (Figure 1b), FA3 (Figure 1c), and FA4 (Figure 1d). The PLA_2_ fractions were checked for homogeneity using SDS-PAGE under reducing (R) and non-reducing (NR) conditions. Under reducing conditions, the electrophoretic patterns of the three fractions were similar, with molecular masses ranging from 15 to 16 kDa. However, in non-reducing conditions, the electrophoretic profile of the FA1 and FA3 fractions showed two smear bands spanning from ~40 to 16 kDa, with a higher staining intensity in the region of ~40 kDa in FA1 (Figure 1e), and a stronger 16 kDa band in FA3 (Figure 1f), suggesting the presence of dimeric forms of these proteins. A single band of ~15 kDa was stained in the FA4 non-reduced fraction (Figure 1g). The isoelectric profiles of the PLA_2_s were analyzed using bidimensional electrophoresis, with non-linear pH values ranging from 3 to 10, showing important variations in the pI values of the proteins. FA1 and FA3 showed basic pIs of 8.4 and 8.2 (Figure 1h,i), while FA4 presented a pI of 5.1, characteristic of acidic PLA_2_s (Figure 1j), respectively. The FA1 fraction presented an extra spot of the same pI around 40 kDa, even in the presence of reducing and alkylating agents that may correspond to low levels of contamination with SVMPs further detected via MS.

**Figure 2:**
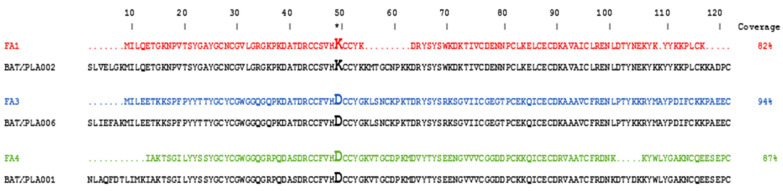
Sequence alignment of identified peptides with complete *B. atrox* PLA_2_ sequences. Peptides of FA1, FA2, and FA3 identified using mass spectrometry were aligned against PLA_2_ sequences previously characterized in *B. atrox* venom using the CLUSTAL W multiple alignment package. Dots represent the uncovered regions, positions not elucidated by MS/MS, of BATXPLA002 (Accession number JAV01882.1), BATXPLA006 (Accession number JAV01878.1), and BATXPLA001 (Accession number JAV01883.1) by peptides identified in FA1, FA3, and FA4, respectively. Asterisk indicates the amino acid residue at 49 position (*).

The bands corresponding to the three isolated fractions were cut off the gel and subjected to mass spectrometry for protein identification. Figure 2 shows the peptides identified in each fraction aligned to the best-matched sequences characterized in a previous transcriptome analysis of venom glands from five *B. atrox* specimens [2]. FA1 peptides aligned with 82% of the BATXPLA002 sequence, including the segment comprising the calcium-binding motif, highlighting the substitution of the canonic aspartic acid at position 49 for lysine. FA3 and FA4 presented 94% and 87% coverage with BATXPLA006 and BATXPLA001, respectively, and both sequences presented aspartic acid at position 49. The canonic cysteine residues were conserved at the same positions in the three isoforms and the degree of sequence identity between the isolated PLA_2_s ranged from 46 to 59%.

### 2.2. Functional Differences in Three PLA_2_ Fractions Obtained from B. atrox Venom

**Figure 3:**
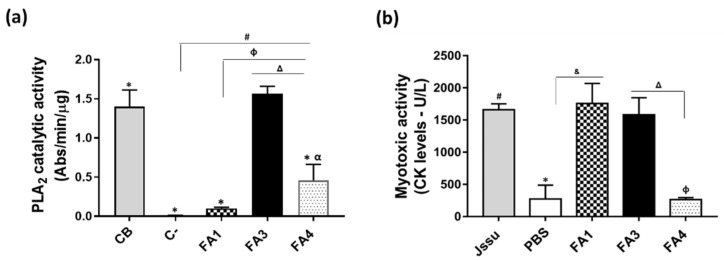
Characterization of catalytic and myotoxic activities of isolated PLA_2_s. The PLA_2_ catalytic activity (**a**) was determined using NOBA as substrate and 0.3 µg/20 μL of PLA_2_ samples or crotoxin B (CB), as a positive control or 20 μL of standard buffer (C−, negative control). The absorbance was measured at 425 nm, and the results are expressed as Abs/min/µg of protein. Symbols indicate differences that are statistically significant for *p* ≤ 0.005: (*) CB compared to FA1, FA4, and negative control; (#) FA3 compared to FA4 fraction and negative control; (Ф) FA3 compared to FA1 fraction; (∆) FA3 compared to FA4 fraction. (α) FA4 fraction compared to negative control. Myotoxic activity (**b**) was measured via CK levels in the serum of mice (*n* = 5), after intramuscular injection in the gastrocnemius muscle of 25 µg of the PLA_2_s, dissolved in 50 µL of PBS. Control groups received injections containing 50 µL of PBS only (negative control) or 50 µg/50 µL of *B. jararacussu* venom (Jssu) as a positive control. After 3 h, blood samples were collected and the CK levels were assayed in serum samples using a commercial kit CK-UV (Bioclin) and with CK levels expressed in U/L. Symbols indicate differences that are statistically significant for *p* ≤ 0.005: (*) PBS group compared to Jssu; (#) Jssu group compared to FA4; (&) FA1 group compared to PBS; (Ф) FA4 compared to FA1; (Δ) FA3 group compared to FA4. Results are expressed as mean ± SD or mean ± SEM of three independent experiments for PLA_2_ activity and myotoxic activity, respectively.

The typical activities of snake venom PLA_2_s were investigated in the isolated fractions, starting with the analysis of catalytic and myotoxic activities. The catalytic PLA_2_ activity was investigated using a synthetic substrate (NOBA), and as shown in Figure 3a, the FA3 fraction presented a higher catalytic activity, comparable to that observed with Crotoxin B used as a positive control. The hydrolysis of the substrate by FA4 was significantly lower than by FA3, and the FA1 fraction had a very low catalytic activity (Figure 3a), in agreement with the literature [17] that aspartic acid at position 49 is important for catalysis. On the other hand, the myotoxic activity of the fractions, measured through CK levels in the serum of mice injected with the proteins, was highly induced by the basic FA1 and FA3 fractions, despite the presence of catalytic activity or the substitution Asp-49-Lys. FA4 was not myotoxic, and induced CK levels similar to the negative control (PBS) (Figure 3b).

**Figure 4:**
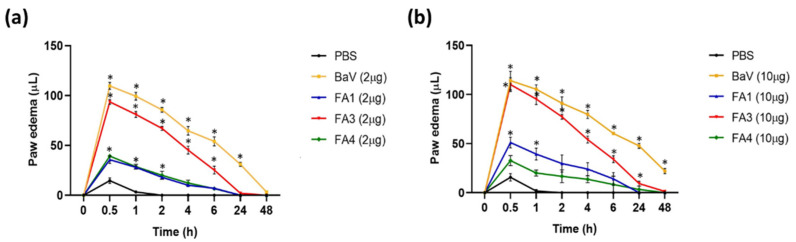
Evaluation of edema formation after intraplantar injection of the PLA_2_s. Groups of five mice were injected subcutaneously into the footpad of the right hind paw with samples of the fractions (FA1, FA3, and FA4) containing 2 µg (**a**) or 10 µg (**b**) dissolved in 30 µL sterile PBS. Control groups were injected with the same volume of PBS (negative control) or *B. atrox* venom (BaV) at the same doses as fractions. The edematogenic activity was evaluated at different times: 0 min (before the treatment), 30 min, 1, 2, 4, 24, and 48 h (after treatment). Edema was estimated using the increase in paw thickness after the injection of the fractions, using a plethysmometer to measure the difference in volume displaced (µL). The data represent the mean ± SEM of three independent experiments. Symbol (*) indicates differences statistically significant for *p* ≤ 0.005 compared to the PBS control group.

Next, we evaluated the edematogenic activities of the PLA_2_s in groups of mice injected s.c. in the right hind paw with 2 µg or 10 µg of isolated fractions. The paw swelling was measured before the treatment (0 min) and 0.5, 1, 2, 4, 6, 24, and 48 h after the injection. As presented in Figure 4, paw thickness increased rapidly after the injection of all PLA_2_s at both concentrations. Intraplantar injections of FA1, FA3, and FA4 fractions caused time-dependent mice hind-paw edema, compared to that observed in the PBS-injected animals, with the highest peak observed at 30 min post-inoculation and sustained for at least 4 h. After 48 h, the volumes of the animals’ paws were restored to the baseline levels. Although all fractions induced edema, the FA3 fraction presented a substantially higher activity than FA1 and FA4 fractions, in practically all the time intervals, with levels comparable to the edema induced by the whole venom. Furthermore, the edema induced by 2 µg of the fractions FA1 and FA4 were similar (Figure 4a), while the subcutaneous injection of 10 µg of FA1 promoted a slightly higher increase in the paw edema in the animals when compared to the FA4 fraction (Figure 4b).

**Figure 5:**
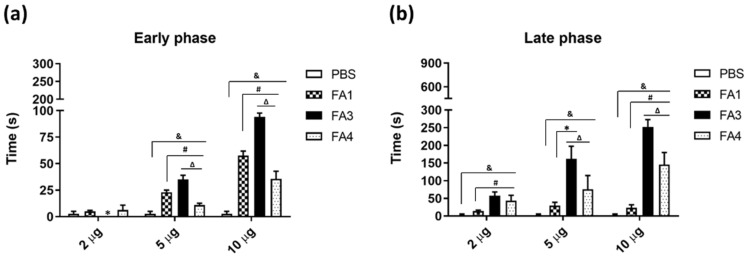
Nociceptive response in mice after intraplantar injection of the PLA_2_s. Fractions (2, 5, or 10 µg/30 µL) were dissolved in sterile PBS injected (i.pl.) into the right hind paw of mice (*n* = 5). Control groups were injected with 30 μL of sterile PBS only. The nociceptive response, evaluated by the reactivity of animals to lick the injected foot, was scored in seconds: (**a**) response in the early or neurogenic phase (from 0 to 5 min after the injection); (**b**) late or inflammatory phase (from 15 to 30 min after the injection). The data represent the mean ± SEM of three independent experiments. Symbols indicate differences statistically significant for *p* ≤ 0.005: (&) PBS group compared to FA1, FA3, and FA4; (#) FA1 group compared to FA3 and FA4; (∆) FA3 group compared to FA4; (*) FA3 group compared to FA1.

The nociceptive effect was then evaluated via the intradermal inoculation of different doses of FA1, FA3, and FA4 fractions or PBS into the right hind paw of the mice. The duration time of the animal licking the injected paw was recorded from 0 to 5 min (neurogenic phase), and from 15 to 30 min (inflammatory phase) after the inoculation with the toxin. As shown in Figure 5a, in the neurogenic phase, the animals injected with the lowest dose (2 µg) of toxins did not show any difference in comparison to the PBS group. At the higher doses (4 and 10 µg) the FA1 and FA3 fractions induced significantly more prolonged nociception than the FA4 group. The FA3 fraction presented the highest nociceptive activity at the neurogenic phase. During the inflammatory phase, the animals that received all doses of FA3 and FA4 showed a more prolonged nociceptive behavior, while the nociception induced by the FA1 fraction was significantly lower than the other groups, in all doses tested (Figure 5b).

**Figure 6:**
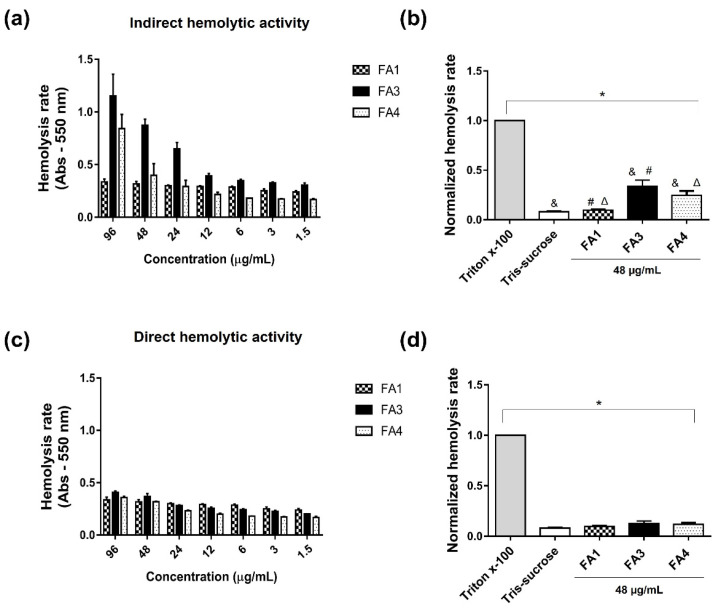
Evaluation of the hemolytic activity induced by the PLA_2_s. The hemolytic activity of the PLA_2_s was tested using washed human red blood cells. Serially diluted concentrations (96, 48, 24, 12, 6, 3, and 1.5 µg/mL) of the fractions FA1, FA3, and FA4 were added to 100 µL of erythrocyte suspensions (1 × 10^8^) and incubated for 1 h with gentle mixing at 37 °C. Next, plates were added with 50 µL of human serum of the same donor for the indirect test (**a**,**b**), or with 50 µL of Tris-sucrose buffer for the evaluation of indirect hemolytic activity (**c**,**d**), and incubated under the same experimental conditions. For the controls, 50 µL of 3% Triton X-100 (positive) or 50 µL of Tris-sucrose (negative) were used. The samples were centrifuged (200× *g* for 5 min, 25 °C), and the absorbance of the supernatant was measured in a spectrophotometer at 550 nm. The experiments were performed in triplicate, and the results represent the mean ± SD of three independent experiments. Symbols indicate differences that are statistically significant for *p* ≤ 0.005: (*) positive control compared to Tris-sucrose, and to FA1, FA3, and FA4 fractions; (&) Negative control (Tris-sucrose) compared to FA3 and FA4; (∆) and (#) FA1 compared to FA3 and FA4, respectively.

Knowing that some PLA_2_s from snake venoms may induce hemolysis, the next step was to evaluate the effect of PLA_2_s on human erythrocytes, in the presence of human serum for the indirect test, or only Tris-Sucrose buffer for the direct test. The results showed that the FA3 and FA4, but not the FA1 fractions presented moderate indirect hemolytic activity in human red blood cells when compared to the negative control group (Figure 6a,b). Neither of these toxins at different concentrations produced the direct hemolysis of human erythrocytes (Figure 6c,d). This finding suggests that PLA_2_s induce hemolysis in red cells only in the presence of serum characterizing indirect hemolytic activity.

**Figure 7:**
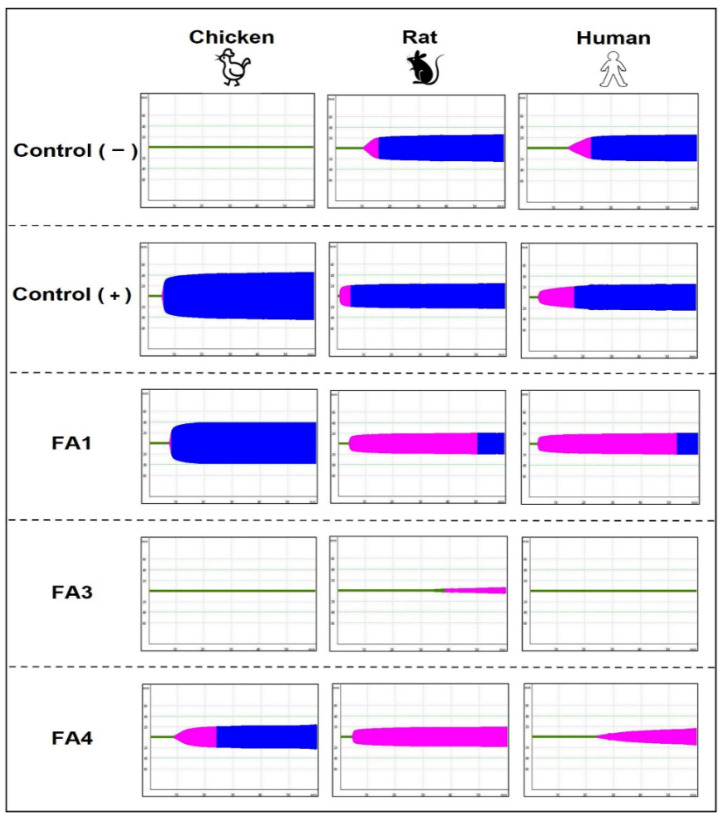
Anticoagulant action of the PLA_2_s on the plasma of birds, rodents and humans. The anticoagulant activity was assessed on recalcified and activated plasmas of chicken, rat and humans. Samples containing 0.5 µg/10 µL of each toxin were pipetted onto specific cups containing 20 µL of 20 mM CaCl_2_ and 60 µL of the coagulation activator ellagic acid. Next, 240 µL of plasma were added to the reaction mixture, and the clotting time (CT), the clot formation time (CFT) and maximum clot firmness (MCF) were recorded by thromboelastometry for 1 hour. The same volume (10 µL) of PBS with or without ellagic acid-based activator of coagulation, were used as positive and negative controls, respectively. The green line (CT) represents the beginning clot formation; pink and blue backgrounds represent clot formation time (CFT) and maximum clot firmness (MCF), respectively. The data shown are representative of three experiments.

Furthermore, the interference of the isolated PLA_2_s on the blood coagulation system was investigated using different plasmas (human, rat, and chicken) and the clotting times were recorded by rotational thromboelastometry, evaluating the main parameters: clotting time (CT), the clot formation time (CFT), and the maximum clot firmness (MCF). As observed in Figure 7 and Table 1, spontaneous plasma coagulation, using PBS as the negative control, was observed in rat and human plasmas, but chicken plasma remained unclotted during the experimental time, clotting only after the addition of the ellagic acid-based activator of coagulation (positive control). Concerning the isolated PLA_2_s, the FA1 fraction reduced the coagulation parameters induced by the ellagic acid-based activator in the three plasmas tested, substantially increasing the CFTs of human and rat plasmas, and reducing the MCF values of all plasmas compared to the positive control. The FA4 fraction induced a slight increase in the CT, prolonging the CFT and reducing the MCF parameter in all plasmas. The FA4 anticoagulant activity was even more evident in human plasma, significantly altering the main parameters evaluated when compared with samples incubated only with the ellagic acid. The FA3 fraction was the most potent anticoagulant, maintaining human and bird plasmas unclotted for the period of 1 h and significantly reducing the coagulation of rat plasma when compared to the results obtained in tests with spontaneous coagulation or in the presence of the coagulation activator. Therefore, all fractions presented degrees of anticoagulant activity, which was more prominent in fraction F3.

### 2.3. Differences in Recognition and Neutralization by Antivenoms of PLA_2_s from B. atrox Venom

**Figure 8:**
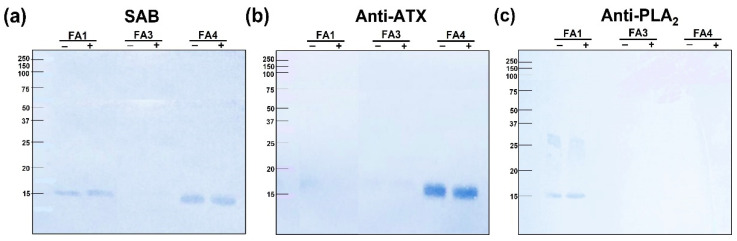
Antigenic reactivity of the PLA_2_s with different antivenoms, evaluated via immunoblotting. Samples containing 10 µg of each PLA_2_ were separated in a 15% SDS-PAGE gel under reducing conditions using DTT (+) and non-reducing (−) conditions. The proteins were transferred onto nitrocellulose membranes, which were blocked and incubated with (**a**) SAB (1:1000); (**b**) anti-ATX (1:100); or (**c**) anti-PLA_2_ (1:40). Immunoreactivity was detected using IgG conjugated to peroxidase of anti-horse (SAB), anti-rabbit (anti-ATX), or anti-mice (anti-PLA_2_) antivenoms, diluted 1:1000. The reactive bands were developed with 0.05% 4-chloro-1-naphthol in 15% (*v/v*) methanol, in the presence of 0.03% (*v/v*) H_2_O_2_.

Considering the structural and functional differences of the three PLA_2_s isolated from *B. atrox* venom, the next step was to evaluate their reactivity and neutralization by the commercial Bothrops Antivenom (SAB) in comparison to a homemade *B. atrox* antivenom (anti-ATX) raised in rabbits, and antibodies against BthTX-I, a Lys-49 PLA_2_ homolog isolated from *B. jararacussu* venom (anti-PLA_2_), raised in mice. As shown in Figure 8, the Western blotting analysis revealed a remarkable antigenic variation between PLA_2_s from *B. atrox* venom. The commercial antivenom (SAB) recognized FA1 and FA4 bands, both under reducing and non-reducing conditions (Figure 8a). The antiserum against-BthTX-1 (anti-PLA_2_) recognized only FA1, presenting a weak reactivity with both the monomeric and dimeric forms (Figure 8b). The anti-B. atrox venom serum (anti-ATX) strongly reacted with the FA4 fraction, but the FA1 fraction showed only a weak reactivity (Figure 8c). Interestingly, none of the tested antivenoms were able to recognize the FA3 fraction, which, besides a potent myotoxic effect, also presented high edematogenic and anticoagulant activities.

**Figure 9:**
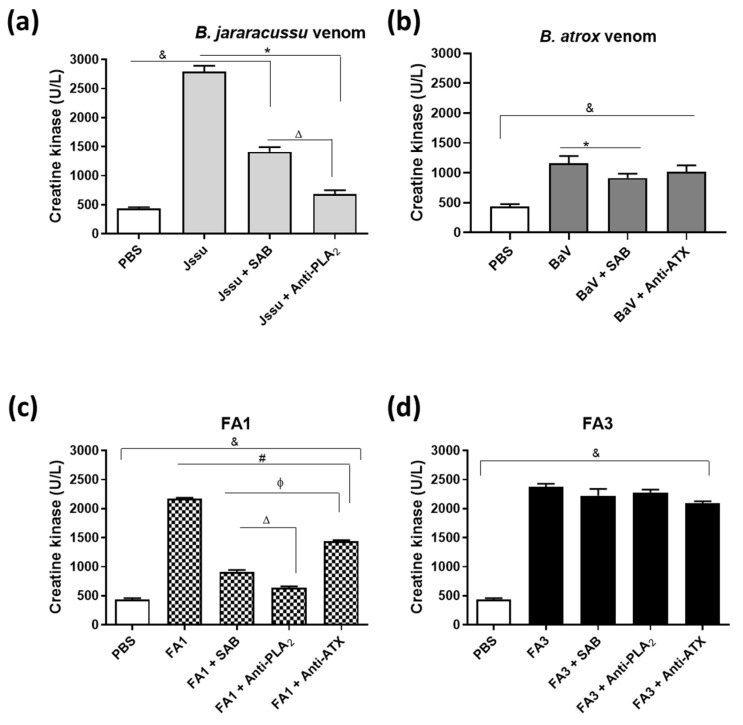
Neutralization by antivenoms of the myotoxic activity induced by FA1 and FA3 PLA_2_ fractions. The neutralizing efficacies of the different antivenoms (SAB, Anti-ATX, and Anti-PLA_2_) were evaluated in groups of 5 mice, intramuscularly (i.m.) injected into the gastrocnemius muscle, with samples containing 25 µg/50 µL of the *B. jararacussu* venom (**a**), *B. atrox* venom (**b**) or the myotoxins FA1 (**c**) or FA3 (**d**), 50 µL of PBS (negative control), previously incubated with SAB, anti-ATX or anti-PLA_2_, as appropriate (0.2 µL of AV/µg of PLA_2_). Three hours after inoculation, blood samples were collected from the mice orbital plexus and centrifuged (2000× *g* for 5 min, 4 °C) to obtain the sera. CK levels of the sera were measured using a commercial kit, CK-UV (Bioclin), and expressed as U/L. The data shown represent mean ± SD of three experiments. Symbols indicate differences statistically significant for *p* ≤ 0.005: (&) PBS group compared to Jssu, and Jssu + SAB; (*) Jssu group compared to Jssu + SAB, and Jssu + anti-PLA_2_; (∆) Jssu + SAB group compared to Jssu + anti-PLA_2_. (&) PBS group compared to Jssu and Jssu + SAB; (*) Jssu group compared to Jssu + SAB, and Jssu + anti-PLA_2_; (∆) Jssu + SAB group compared to Jssu + anti-PLA_2_. (&) PBS group compared to FA1, FA1 + SAB, FA1 + anti-PLA_2_, and FA1 + anti-ATX; (#) FA1 group compared to FA1 + SAB, FA1+ anti-PLA_2_, and FA1 + FA1 + anti-ATX; (ϕ) FA1 + SAB group compared to FA1 + anti-PLA_2_, and FA1 + anti-ATX; (∆) FA1 + SAB group compared to FA1 + anti-PLA_2_. (&) PBS group compared to FA3, FA3 + SAB, FA3 + anti-PLA_2_, and FA3 + anti-ATX.

One of the most relevant activities of snake venom PLA_2_s for human envenomings is their myotoxic activity, present in the FA1 and FA3 fractions. Therefore, we evaluated the residual myotoxic activities of these fractions after their incubation with the commercial and homemade antivenoms (SAB, anti-PLA_2_, and anti-ATX), using as a positive control the neutralization of the same activity of *B. jararacussu* venom. As shown in Figure 9, the group injected with the *B. jararacussu* venom had an increase in the CK serum levels, compared to mice injected only with PBS, and the myotoxicity of this venom had been significantly inhibited by both the commercial antivenom (SAB) and the anti-PLA_2_ (BthTX-1) antibodies (Figure 9a). Concerning the experimental samples, the myotoxicity induced by FA1 was significantly neutralized by all tested antisera, with the SAB and anti-PLA_2_ being the most efficient (Figure 9b), although with a lower efficacy. However, none of the antivenoms neutralized the myotoxic activity induced by the FA3 fraction (Figure 9c).

Table 2 shows a summary of the most important characteristics of each of the toxins, highlighting their differences. These results revealed the structural and functional diversity of the PLA_2_s from *B. atrox* venom, and also a differential degree of antigenicity among the isolated toxins. FA3 was the most active isoform in the different tests performed; however, this fraction was not recognized by antivenoms, bringing some important concerns toward the efficacy of antivenom therapy in human cases of snakebite.

## 3. Discussion

Snakebites are an important public health problem, particularly in rural areas of tropical countries. In Brazil, snakebites affect thousands of patients every year and the highest incidences of envenomings are found in the Amazon region [37], in which *Bothrops atrox* is widely distributed [38] and is the main species associated with the snakebites [37]. The clinical symptoms caused by *B. atrox* venom in patients include local effects such as pain, edema, myonecrosis, spontaneous bleeding at the bite site, and systemic reactions represented mainly by coagulopathies [39,40]. Among the venom composition, snake venom PLA_2_s are frequently associated with most of these symptoms, which depend on the presence and abundance of distinct PLA_2_s isoforms that contribute to the functional variability described for *B. atrox* venom [4]. Recently, we demonstrated the individual variability of toxic effects of *B. atrox* venom samples obtained from 37 specimens using two different animal models: birds and mammals. PLA_2_s were among the components associated with the functional variability, possibly to benefit the capture of different prey [41]. Herein, we extended the information on venom functional variability by analyzing PLA_2_s isolated from *B. atrox* venom on a wide array of toxic activities and most importantly, on their reactivity with antivenoms.

Three PLA_2_s have been isolated and characterized: FA1, FA3, and FA4, which included the most relevant structural forms of PLA_2_s from viper snakes: FA1 is a Lys-49 basic PLA_2_ homologue with undetectable catalytic activity. As with other reported Lys-49 PLA_2_s [17,36], FA1 assembles in homodimers with subunits that strongly interact through non-covalent bonds. FA3 is also a basic PLA_2_, but it is an Asp-49 isoform that fully expresses its catalytic activity. FA4 is an acidic, catalytic, Asp-49 isoform. Some PLA_2_s have already been isolated from *B. atrox* venom. Furtado et al. [35] isolated one Lys-49 isoform without catalytic activity (BaTX-I), and two catalytically active Asp-49 isoforms, one of acidic pI (BaPLA_2_) and one a basic toxin (BaTX-II), all capable of inducing inflammatory effects. Additionally, there are reports of isolation of BatroxPLA_2_, an acidic PLA_2_ with a high catalytic activity that induced the release of IL-6, PGE_2_, and LTB_4_ from murine macrophages in culture [32]; BaPLA_2_M-I, a Lys-49 homologue that induces edema, local myonecrosis, and IL-6 production in vivo [33]; BaPLA(2)I and BaPLA(2)III, two myotoxins with catalytic activity capable of degranulating mast cells in vitro and causing edema and myonecrosis in vivo [34]; and BaMtx, a Lys-49 myotoxic homologue with therapeutic potential [36]. Unfortunately, most of these proteins lack primary structure characterization, except BaMTx, which shares 85% identity with FA1. On the other hand, the functional tests included in the former studies were mostly related to the induction of myonecrosis and the inflammatory reaction, impairing a more direct comparison with the functional activities of the PLA_2_s described in this study.

Myonecrosis is the major effect induced by PLA_2_s from *Bothrops* venoms. Accordingly, FA1 and FA3 were strongly myotoxic, increasing the CK serum levels in injected mice, while FA4 did not display such activity, resulting in CK serum levels comparable to the ones found in the negative control group. In general, the results reported above agree with reports from the literature [17], as myotoxic PLA_2_ are mostly the basic isoforms. Interestingly, regardless of the ability to hydrolyze phospholipids, both Asp-49 and Lys-49 isoforms cause myonecrosis to comparable extents [42], as observed in this study with the FA3 and FA1 fractions. Since Lys-49 isoforms are weakly catalytic, their membrane-damaging myotoxic activities have been attributed to the C-terminal region containing basic and hydrophobic residues, which have been strongly associated with the ability to interact and penetrate the lipid bilayer [43]. In Asp-49 PLA_2_, the catalytic activity participates in the myotoxic effect, as its inhibition reduces myonecrosis to varying degrees of residual toxicity; however, the residual activity demonstrates the involvement of additional membrane disturbance mechanisms outside of the catalytic site regions [27,44].

We also investigated the edematogenic and nociceptive activities of the isolated isoforms in a murine model. The three fractions were capable of inducing edema in the paws of mice, and FA3 was more potent than FA1 and FA4. Regarding the nociceptive activity, in the first five minutes after the inoculation (neurogenic phase), FA1 and mainly FA3 induced greater nociception. Interestingly, after this time and until the end of the test (inflammatory phase), FA1 maintained the same profile, with no apparent increase, while FA4 and FA3 induced greater nociception in the animals (Figure 5a,b). These results suggest that the catalytic activity is implicated in edematogenic and inflammatory nociception induced by snake venom PLA_2_s, and is in agreement with previous reports with other Lys-49 homologues [36]. In general, the edema induced by PLA_2_s from viperid venoms promotes an early increase in the vascular permeability and release of inflammatory mediators, which act synergistically to cause inflammatory events [23,45], and both the Lys-49 and Asp-49 isoforms are capable of inducing local inflammation, despite of their catalytic differences [42]. However, considering the higher extent of the inflammatory effects induced by the catalytic forms of FA3 and FA4, it is reasonable to suggest that events such as edema and nociception may include key steps in the cascade of inflammatory mediators, in which the hydrolysis of phospholipids (PPLs) plays an important role. The inflammatory responses to venoms of *Bothrops* snakes are part of the local manifestations of human envenomations, and they contribute to the evolution of extensive tissue damage that may lead to permanent disabilities [46] through a set of events including increased vascular permeability, edema, hyperalgesia, and the activation/infiltration of immune cells [42]. In this study, after 48 h of experiment, the edematogenic actions of all PLA_2_s fractions were reduced to the basal level, while residual edema remained in the group injected with the whole venom, which may be associated with the action of SVMPs, the more abundant toxins in the *B. atrox* venom [4]. Consistent with that, several studies demonstrate that SVMPs induce inflammatory events [47,48]; however, the local lesions induced by venoms of *Bothrops* snakes have been mostly attributed to the synergistic action of SVMPs and PLA_2_s [49,50]. Thus, the results shown here with the myotoxic, edematogenic, and nociceptive actions of FA1, FA3, and FA4 indicate that these PLA_2_s contribute to the local symptoms of the envenomings, depending on their presence and prevalence in the venoms, or even as a synergistic factor to SVMPs.

In addition to the important contributions of PLA_2_s to venom-induced local effects, they also contribute to hemostatic disorders characteristic of the systemic effects of the Bothrops envenomings [51] by additive or synergistic effects with CTLs, SVSPs, and SVMPs [52,53]. Thus, we also investigated the effects of the isolated PLA_2_s on hemolytic activity (direct and indirect) using human erythrocytes, and the anticoagulant effects on plasmas from rats, chickens, and humans. Consistent with the presence of catalytic activity, only FA3 and FA4 presented indirect hemolytic activity, while FA1 had no hemolytic effect. Testing the anticoagulant activity, FA3 proved to be the most potent, followed by FA4, while FA1 showed only minor anticoagulant action. Interestingly, FA3 had a strong effect in all types of plasmas, although a slightly smaller effect on rat plasma; on the other hand, FA4 was more potent in human than rodent plasma, but did not significantly affect avian plasma. Several studies have suggested that the anticoagulant action of snake venom PLA_2_s depends on their enzymatic activities, as the catalytic hydrolysis of PPLs from the platelet surfaces would impair the coagulation cascade [54]. However, the literature also reports that the anticoagulant effect of PLA_2_s can occur via catalysis-independent mechanisms, since some PLA_2_s interact directly with coagulation factors, inhibiting the formation of tenase and/or prothrombinase complexes, key enzymes of the coagulation cascade [31,55]. Herein, the results of the hemolytic and anticoagulant activities correlate with the catalytic actions of the PLA_2_s, as FA3 has the highest enzymatic, anticoagulant, and hemolytic activities. However, it is important to note that even the low catalytic Lys-49 FA1 presented some degree of anticoagulant activity, particularly altering the clot firmness of all tested plasmas. Regarding human plasma, the differences could be related to a possible action of FA3 on some enzymes of the coagulation cascade, as reported for other anticoagulant PLA_2_s [56]. On the other hand, the differences observed in rat and chicken plasmas, mostly with FA4, seem more related to intrinsic differences in the coagulation systems of mammals and birds [57,58] and could represent an adaptive trait, as it was suggested that the expression of the PLA_2_ isoforms would reflect environmental evolutive adaptations related to prey availability [59].

Venom composition and isoform variability are likely to have fitness consequences for individual snakes [60,61]. Studies of venom variation in an ecological context suggest that snake diet could drive selection for the presence of multiple isoforms of a toxin present within the venom of a single species or individual [62]. In agreement with this hypothesis, the evolution of snake venom PLA_2_ genes indicates they have diversified, with the majority of gene duplications occurring towards the tips of the species phylogeny and involving the evolution of major functions such as neurotoxic, anticoagulant, pro-inflammatory, and myotoxic activities [63]. However, it is not yet clear whether the acquisition of functional diversity has arisen as a result of the neofunctionalization or subfunctionalization of ancestral multifunctional activities. Our model strongly supports the neofunctionalization hypothesis, since the fractions are all multifunctional, particularly FA3, which displayed all the activities tested here. This is in agreement with the hypothesis of Malhotra et al. [63] that multifunctional proteins appear more at the tip than the ancestral nodes of the evolution of PLA_2_ genes, and are apparently under diversifying selection. In a more recent analysis, Suranse et al. [64] reported several sites experiencing an episodic diversifying selection for the viperid Asp-49 PLA_2_ group, while very few sites were identified in Lys-49, which could be a consequence of the recent evolution of these forms. This observation could explain the multifunctionality observed in the FA3 fraction in comparison to the FA1 and FA4 fractions shown here. As for our understanding of the evolutionary origins, phylogenetic relationships, and ecological relevance of PLA_2_s, the multifunctional isoforms would be of great advantage for snake fitness. According to this, the data reported here also revealed for the first time remarkable variations in the anticoagulant effects of the PLA_2_s on the plasmas of rodents, humans, and birds suggesting that the pathophysiological processes induced by these PLA_2_s in different animal models, including natural prey, are important for an understanding the ecological roles of non-neurotoxic PLA_2_s from snake venoms. However, future research is required to reconstruct the evolutionary pathways leading to the origins of these diverse functions of viper venom PLA_2_s, to further understand the possible adaptive roles of their variability in *B. atrox* venom, not only concerning the mechanisms underlying the differential toxic effects, but also the digestion of prey tissues.

If, on one side, it is still uncertain whether venom composition have major fitness advantages for individual snakes, on the other side, it is very clear that venom variability is a problem for the treatment of snakebite envenomings. We have recently shown that the abundance of particular isoforms of different families of toxins in the venom of *B. atrox* specimens correlates with the severity of some symptoms of patients bitten by such snakes [65]. Additionally, the diversity of metalloproteinases in the venoms from *B. atrox* snakes from specific habitats impairs the neutralization of venom coagulotoxic activity *in vitro*, and using experimental models [66,67]. Thus, our last approach was to evaluate the reactivity of the isolated PLA_2_s with three different antivenoms: the commercial antivenom used to treat patients bitten by *Bothrops* snakes (SAB-Bothrops Antivenom), a home-made experimental antivenom raised in rabbits via immunization with *B. atrox* whole venom (Anti-ATX), and one raised via the immunization of mice with Bth TX-I, a Lys-49 PLA_2_ myotoxin isolated from *B. jararacussu* venom (Anti-PLA_2_). The reactivity of these sera with the isolated PLA_2_s was evaluated via Western blotting, with distinct results. Only FA4 and FA1 were recognized by the commercial antivenom; FA1 was also revealed as a faint band by the anti-PLA_2_ and anti-ATX antibodies, while FA4 was not recognized by anti-PLA_2_, but was strongly reactive with anti-ATX antibodies. In this test, FA3 was not recognized by any of the antivenoms tested. A similar result was obtained when the same antivenoms were used for the neutralization of the myotoxic activity induced by the basic fractions FA1 and FA3. Only FA1 was neutralized by the antivenoms, more efficiently by Anti-PLA_2_, followed by the commercial antivenom. However, the potent myotoxic effect of FA3 was not significantly reduced by any of the tested antivenoms.

It is currently accepted that the treatment of snake bites is critically dependent on the ability of antivenoms to reverse the array of venom-induced pathological symptoms via direct neutralization of venom toxins [13]. In Latin America, commercial antivenoms are produced using venoms from a limited number of species as immunization protocols. The selection of the venoms varies according to the species distribution and availability in the countries of each manufacturer. Most antivenoms are safe and effective, but inter-and intraspecific functional variability may result in the clinical variability of envenomation deserving the greatest consideration, since bites by specific populations may require different treatments [68,69]. Considering this limitation, several groups attempt to evaluate the cross-reactivity range and efficacy of commercial or experimental antivenoms to independent groups of toxins, both with decomplexed venoms by antivenomics [70] and with isolated toxins. Zamúner et al. [71] showed that the SAB, produced by Instituto Vital Brazil, only partially neutralized the myotoxic activity induced by five venoms from *Bothrops* species, including *B. jararacussu*, whose venom makes part of the immunization pool used to produce this antivenom [71]. Considering the neutralization of *B. atrox* venom, antivenoms showed impaired immunoreactivity towards PLA_2_ and PI-SVMP molecules according to antivenomics [9] and ELISA [67], and presented a limited neutralization of Factor X activating SVMPs [66].

Fortunately, the cases discussed above are not the general rule. Several studies have shown the efficacy of commercial antivenoms in neutralizing toxic activities induced by *B. atrox* venom, including hemorrhagic and lethal activities [4,10,67]. Moreover, Pardal et al. [39] performed a clinical trial of the commercial and one experimental antivenom, for the treatment of snakebites in the Brazilian Amazon, where *B. atrox* is causative of most accidents. The authors reported that both antivenoms were equally effective in reversing all signs of envenoming [39]. Nevertheless, as discussed above, the composition of the *B. atrox* venom is variable, and even considering an exception, the observations reported in the present study should be taken with concern. FA3 was not recognized or neutralized by the antivenoms. This fraction is found with relative abundance in several individual venoms from *B. atrox* [41], and displayed activities that amplify both the local and systemic symptoms observed in patients bitten by *B. atrox*. Thus, the identification of a venom toxin evading neutralization by the antivenoms may impair the efficiency of the antivenom therapy.

## 4. Conclusions

As a consequence of the intrinsic mechanisms of gene duplication and diversifying selection involved in the evolution of snake venom components, multigene toxin families are present in the presently occurring species, and result in the complex arsenal that comprises snake venoms. We show here a clear example of how these genes that are present in the same species code for isoforms with multiple and functionally distinct activities, resulting in different strategies for prey subduing. Unfortunately, these different toxic isoforms are also damaging factors in human accidental envenomations by the snake, and as shown here, they are not equally neutralized by the antivenoms. In this vein, our findings showing the functional diversity and differential reactivity with antivenoms of PLA_2_s from *B. atrox* venom provide new and important insights into the relationship between venom variability, pathophysiological effects, and antivenom efficacy.

The lack of reactivity with different antivenoms and the poor neutralization of relevant toxic activities, as observed with the myotoxicity induced by FA3 fraction, reinforce the need for special attention from the clinical point of view and the constant surveillance of antivenom efficacy by its manufacturers. In this way, the elucidation of the molecular basis underpinning the antigenic diversity of snake venom PLA_2_s could be a useful tool for the improvement of antivenoms to treat the human envenomings caused by *B. atrox*.

Concluding, a broad knowledge of the ecology and evolution of snakes, and their venom composition and functional studies of isolated forms are important practices that contribute to the improvement of the antivenoms currently available to treat snakebites.

## 5. Materials and Methods

### 5.1. Ethical Statement for the Use of Experimental Animals and Human Samples

For protocols using experimental animals, procedures were approved by the Committee for Animal Research of the Butantan Institute (CEUAIB, protocol number 13710-14, date: 14 September 2017). Male Swiss mice (18–20 g), and male Wistar rats (400–450 g), provided by Instituto Butantan (São Paulo, Brazil), were kept in ventilated cages, with controlled temperature (25 °C), and 12 h light/dark cycles. The animals received standard feed and water ad libitum. White adult male leghorn chickens (2–3 kg) were obtained from commercial breeding and kept at 22 °C with water and commercial feed ad libitum. Samples of human blood were obtained from healthy volunteers following the Brazilian Committee for Human Research instructions (CAAE: 89499218.8.0000.5377). Human and rat blood were collected using vein puncture in the presence of 0.9% sodium citrate; and plasma was used for the anticoagulation assays, and the red cells for hemolytic activity. Citrated plasma of chicken was collected according to Prezoto et al. [72].

### 5.2. Venoms and Antivenoms

*B. atrox* venom was obtained by milking 37 adult specimens of *B. atrox*, male and female, captured in four different habitats (forest, pasture, recently degraded, and floodplain) west of Pará State—Brazil, under license (SISBIO No. 33098-3). After collecting the snakes, the crude venoms were extracted individually and then pooled according to the habitats, centrifuged to remove impurities, lyophilized, and stored at −20 °C until use. Equal amounts of the venoms from each habitat were used to prepare the pool of venoms used to isolate the PLA_2_s. The commercial Bothrops antivenom (SAB) (batch 130577) was produced by Instituto Butantan, São Paulo—Brazil, from horses immunized with a venom mixture containing *Bothrops jararaca* (50%), *Bothrops neuwiedi* (12.5%), *Bothrops alternatus* (12.5%), *Bothrops moojeni* (12.5%), and *Bothrops jararacussu* (12.5%) venom. The SAB is composed of purified F(ab)_2_ fragments of immunoglobulins in liquid form, at 10 mg/mL. The neutralizing potency stipulated by the manufacturer is 1 mL of antivenom for 5 mg of the reference venom of *Bothrops jararaca*. Two other polyclonal antibodies were used: the IgG fraction isolated from an antiserum against *B. atrox* venom (anti-ATX, at 1 mg/mL) produced in rabbits according to [73], and the IgG fraction isolated from an antiserum against a myotoxin (BthTX-I) isolated from *B. jararacussu* venom (anti-PLA_2_, at 0.4 mg/mL), produced in mice according to Moura da-Silva et al. [74].

### 5.3. Isolation of the PLA_2_s Using Reverse-Phase High-Performance Liquid Chromatography (RP-HPLC)

PLA_2_s were purified from *B. atrox* venom via RP-HPLC, using an RP100 C-18 column (250 mm X 4.6 mm, 10 µm particle size, Vydac) coupled to an LC 20–AT HPLC system (Shimadzu, Japan) as previ ously described [67], with few modifications. In brief, venom samples containing 10 mg were dissolved in 500 μL of trifluoroacetic acid (TFA) and centrifuged at 18,400× *g* for 10 min, at 25 °C. Next, the supernatant proteins were applied to the C18-column and eluted with a constant flow of 2 mL/min. For this, a linear gradient of solution A (0.1% TFA in H_2_O) and solution B (100% acetonitrile) was used, as follows: 5% of solution B over 0–10 min, 5–15% B over 10–30 min, 15–45% B over 30–150 min, 45–70% B over 150–170 min, 70–100% B over 170–180 min, and 100% B over 180–190 min. The chromatographic run was monitored at 214 and 280 nm, and the fractions were collected manually and dried in a vacuum concentrator (SpeedVac, Savant, Farmingdale, NY, USA). The fractions of interest (FA1, FA3, and FA4) were collected according to their retention times (RT), resuspended in 1 mL of solution A, and rechromatographed separately, using the same protocol. Each purification cycle consisted of at least 10 chromatographic runs. After the last chromatographies, each fraction was lyophilized and stored at −80 °C until use. The protein concentration was evaluated spectrophotometrically at 280 nm, using a SpectraMax^®^ M2 (Molecular Devices, Sunnyvale, CA, USA), and confirmed using the Bradford method [75].

### 5.4. Gel Electrophoresis

The homogeneity of the fractions was evaluated via SDS-PAGE using 15% polyacrylamide gels, as described by [76]. Samples (10 µg) were reduced with dithiothreitol (DTT) and denatured in an SDS-loading buffer containing 0.125 M Tris-HCl, pH 6.8, 10% glycerol, 2% SDS, and 0.001% bromophenol blue. They were boiled for 5 min, loaded, and run at a constant 35 amp/180 V. Next, the proteins were visualized by staining with Coomassie brilliant blue R-250, followed by destaining in a solution containing 30% ethanol and 10% acetic acid. The molecular masses of the proteins were calculated using a comparison with the molecular mass standard MW (GE Healthcare, Pittsburgh, PA, USA), which consisted of the following: phosphorylase b (97 kDa), bovine serum albumin (66 kDa), ovalbumin (45 kDa), carbonic anhydrase (30 kDa), trypsin inhibitor (20.1 kDa), and α-lactoalbumin (14.4 kDa). The pIs of the isolated proteins were checked using two-dimensional electrophoresis (2D-PAGE) with an Ethan IPGPhor TM (GE Healthcare), according to the manufacturer’s instructions. Briefly, samples (25 μg) were dissolved in 125 µL of rehydration solution containing 7 M urea, 2% (*w/v*) CHAPS, 40 mM DTT, 0.5% (*v/v*) pharmalyte, 2 M thiourea, and 0.002% (*w/v*) bromophenol blue, and incubated with commercial 7 cm precast strips for isoelectric focusing (IEF), with a linear range of pH 3–10 for 12 h at room temperature. The first dimension IEF was performed using the following protocol: 300 V/30 min, 1000 V/30 min, 5000 V/1.20 h, and 5000 V/25 min. Before the second dimension, the precast strips were equilibrated with reduction buffer containing 75 mM Tris/HCl, pH 8.8, 6 M urea, 30% (*v/v*) glycerol, 2% (*w/v*) SDS, 0.002% (*m/v*) bromophenol blue, and 10 mg/mL DTT for 15 min. Next, strips were reduced and alkylated using the same solution plus iodoacetamide (25 mg/mL) for an additional 15 min, and analyzed via SDS-PAGE with 15% polyacrylamide. The spots were visualized using Coomassie Brilliant Blue staining. Gel images were scanned using an Image Master LabScan 5.0 with a resolution of 300 dpi. Images were analyzed with the software Platinum 2D 7.0 (GE Healthcare) for the determination of relative molecular mass and isoelectric point (pI) of the spots.

### 5.5. Identification of the PLA_2_s via Mass Spectrometry

Protein bands of interest were excised from polyacrylamide gels, rehydrated, and ~10 µg were submitted to reduction with 10 mM dithiothreitol (DTT) in 0.1 M ammonium bicarbonate, and alkylation with 50 mM iodoacetamide in 0.1 M ammonium bicarbonate (both at room temperature for 30 min). The samples were then digested overnight at 37 °C with 1 µg trypsin in 50 mM ammonium bicarbonate. The samples were acidified with acetic acid to stop digestion, and were then spun down. The supernatant was evaporated to 20 µL for liquid chromatography–mass spectrometry (LC-MS) analysis. The tryptic digests were desalted using an Empore C18-SD 4 mm/1 mL column (Supelco, UK) and subjected to reversed-phase nanochromatography coupled to nanoelectrospray high-resolution mass spectrometry for peptide analysis (nanoLC Easy-LTQ Orbitrap Velos-ETD, Thermo Fisher Scientific, Waltham, MA, USA). Tandem mass spectra were processed and searched against an in-house database composed of the full-length precursor proteins predicted from the transcriptomes of five specimens of *B. atrox* [2], using the search tools Mascot (Matrix Science, London, UK; version 2.6.2) and X! Tandem (The GPM, thegpm.org; version X! Tandem Alanine (2017.2.1.4)). Peptides were assembled by alignment with the best-matched sequence from the database, using Clustal W [77].

### 5.6. Functional Characterization of the PLA_2_s from B. atrox Venom

#### 5.6.1. PLA_2_s Catalytic Activity

The enzymatic activities of isolated toxins were determined as described previously [78], using 4-nitro-3-octanoyloxy-benzoic acid (_4_N_3_OBA) (Biomol, Plymouth Meeting, PA, USA) as a substrate. In brief, 20 µL containing 0.3 µg of the PLA_2_s were placed in a 96-well plate containing 220 µL standard buffer (10 mM Tris-HCl, 10 mM CaCl_2_, and 100 mM NaCl, pH 8.0). Then, 20 µL _4_N_3_OBA (320 µM in DMSO) was added to the mixture and incubated at 37 °C for 40 min. The catalytic activity was calculated based on the absorbance variation (at 425 nm) as a function of the substrate released per minute/µg of protein (Abs/min/µg). All assays were performed in triplicate, and absorbances were measured in a spectrophotometer (M12 Multiskan EX, Labsystems, Vantaa, Finland). The CB subunit of the crotoxin (CTX) isolated from Crotalus durissus terrificus venom and PBS were used as positive and negative controls, respectively.

#### 5.6.2. Hemolytic (Direct and Indirect) Activity

Direct and indirect hemolytic activities were assayed in 96-well plates as described previously [79]. Blood obtained from human healthy volunteers was collected in tubes containing 0.9% sodium citrate as the anticoagulant, and centrifuged at 200× *g* for 5 min at 25 °C. Plasma was removed and the erythrocyte suspension was washed three times with Tris-sucrose buffer (10 mM Tris/HCL pH 7.4, 250 mM sucrose, 0.8 mM MgCl_2_, and 0.3 mM CaCl_2_). After the last washing step, the supernatant was decanted, and the erythrocytes were counted and diluted at 1:10 in Tris-sucrose buffer. For both the direct and indirect assays, 1 × 10^8^ erythrocytes and different concentrations of the PLAs_2_ (96, 48, 24, 12, 6, 3, and 1.5 µg/mL) were added to a plate in a final volume of 100 µL/well, and incubated for 1 h with gentle agitation at 37 °C. After this period, the red cells were incubated again for 1 h under gentle agitation at 37 °C, with 50 µL of serum/well, assuming that the erythrocytes and serum were obtained from the same donor (indirect test), or incubation was made with 50 µL of Tris-sucrose (direct test). References to 100% and 0% hemolysis were made by incubating a suspension of red cells with 3% Triton X-100 (positive control) and a negative control using only Tris-Sucrose buffer, respectively. Controls and samples were centrifuged (200× *g* for 5 min at 25 °C), and the hemolysis rate was determined via measurement of the released hemoglobin in a spectrophotometer (M12 Multiskan EX, Labsystems, Finland) using the absorbance values of the positive control as 100% lysis at 550 nm.

#### 5.6.3. Evaluation of the PLA_2_s Anticoagulant Activity on Human and Animal Plasmas

The effects of PLA_2_s on blood clotting were investigated in different types of plasma (human, rat, and chicken) using a four-channel ROTEM^®^ system (Pentapharm, Munich, Germany), as described previously [72]. For that, samples (240 µL) of human plasma recalcified with 20 µL of CaCl_2_ (0.02 M), treated only with 70 µL of PBS pH 7.5 in a final volume of 340 µL, were used as negative controls (spontaneous clotting). For the positive controls, recalcified plasma samples were treated with 60 µL of a solution containing TTPa clot (an activator of coagulation containing ellagic acid plus synthetic phospholipids) (Logica Diagnostica, São Paulo, Brazil). In the tests with PLA_2_s, 0.5 µg/10 µL samples were added to specific cups containing 60 µL of ellagic acid, 20 µL of 0.2 M CaCl_2_, and 10 µL of PBS. Next, the plasmas were added, and the effects of the PLA_2_s on the coagulation were evaluated for 1 h, using a graphic record (thromboelastogram) of the main coagulation parameters, CT, CFT, and MCF. CT—Clotting time (s), corresponds to the period from the start of the analysis until the beginning of the clot formation. CFT—Clot formation time (s), is the period after CT, which represents the kinetics of thrombin formation, fibrin polymerization and clot stabilization. MCF—Maximum clot firmness (mm), is the period after CFT, which consists of the maximum amplitude of the clot, due to its stabilization via fibrin polymerization.

#### 5.6.4. Myotoxic Activity

To investigate the myotoxic activity of the PLA_2_s fractions, 25 µg samples were dissolved in 50 μL of PBS pH 7.5 and injected intramuscularly (i.m.) into the gastrocnemius muscle of mice (*n* = 5 per group). Control groups received an identical injection of PBS (50 µL) or 50 µg of *B. jararacussu* venom (Jssu), used as negative and positive controls, respectively. After 3 h, the blood samples were collected via retroorbital puncture into a heparinized capillary and centrifuged (5 min/4 °C at 2000× *g*). The creatine kinase (CK) activity, expressed in units U/L, was determined using a commercial kit CK-UV (BIOCLIN, Belo Horizonte, MG, USA), according to the manufacturer’s instructions. One unit corresponds to the amount of enzyme that hydrolyzes 1 mmol of creatine per min. The results represent the mean ± SD of three independent experiments.

#### 5.6.5. Edematogenic Activity

The edematogenic activity of PLA_2_s was evaluated as described previously [80], with some modifications. Groups of five animals were injected (i.pl.) into the footpad of the right posterior paw, with 2 or 10 µg / 30 µL of the proteins or BaV. Control animals were injected with 30 µL of PBS only. The evolution of the edema was evaluated at different periods (15 min, 0.5, 1, 2, 4, 6, 12, 24, and 48 h) using a plethysmometer (7140 Plethysmometer, Ugo Basile, IT, USA). The paw thickness was assessed before sample injection for basal measurement, and after the injection with the fractions. Paw edema was estimated by calculating the volume difference compared to the basal paw volume for each time-point. The results are expressed as paw volume (in µL) and represent the mean ± SD of three independent experiments.

#### 5.6.6. Assessment of PLA_2_s-Induced Nociceptive Activity

The nociceptive action of the PLA_2_s was evaluated with an adaptation from the formalin test in mice, in which two distinct periods of high licking activity can be identified: an early phase, lasting the first 5 min and a late phase, lasting from 20 to 30 min after the injection of formalin [81]. In our study, noxious stimulus (formalin) was replaced by the PLA_2_s fractions. Before the test, Swiss mice were adapted on a reflecting surface under a glass funnel, 10 min before the PLA_2_s inoculation. Then groups of six mice were injected with samples containing 2, 5, or 10 μg of protein/30 μL PBS in the right hind leg. The left hind leg received an equal volume of sterile saline alone, and served as the negative control. The nociceptive activity was based on the time amount that the animal spent licking the injected paw with the PLA_2_s on the dorsal surface of the paw, toes, or legs. The time was measured in seconds, during 30 min of experimental evaluation, divided into the early or neurogenic phase (0–5 min or 300 s), and the late or inflammatory phase (15–30 min or 900 s).

### 5.7. Reactivity with Antivenoms

Firstly, the reactivity of the PLA_2_s was tested via Western blotting using three different antivenoms (SAB, Anti-B. atrox, and Anti-PLA_2_s). Briefly, the PLA_2_s were subjected to an SDS-PAGE as described above, and then electrotransferred to nitrocellulose membranes (Hybond-ECL-nitrocellulose, Amersham Biosciences, Litle Chalfont, UK), where the reaction with the antibodies occurred. The transfer was performed in a semi-dry system from Bio-Rad (Semi-dry system, Bio-Rad Laboratories, Hercules, CA, USA), using transfer buffer (48 mM Tris, 39 mM glycine, 0.37% SDS, and 20% methanol, for 1 h, at 20 V and 200 mA). Subsequently, the membranes were blocked with 5% skimmed milk in TBS, washed, and incubated under agitation for 2 h, with the primary antibodies applied: SAB (1:1000), Anti-ATX (1:100), or Anti-PLA_2_s (1:100) diluted in 5% skimmed milk in TBS. Following, the membranes were subjected to a new wash cycle and incubated, as appropriate, with antibodies anti-IgG from horse, rabbit, or mice, conjugated with peroxidase (Sigma-Aldrich, St. Louis, MO, USA), diluted 1:1000 in 5% skimmed milk in TBS, for 2 h, at room temperature. After the last washing cycle, the reaction was developed with a chromogenic substrate (4-chloro-1α-naphthol 0.05% (*v/v*) in methanol 15%, in the presence of 0.03% (*v/v*) H_2_O_2_). The enzymatic activity was interrupted by successive washes of the membranes with running water.

### 5.8. Neutralization of the Myotoxic Activity

Samples containing 25 µg of each PLA_2_ dissolved in PBS were incubated individually at 37 °C, under agitation for 30 min, with 15 µL of the three antivenoms at a concentration of 10 µg/mL. Control groups were inoculated with 50 µL of PBS (negative control), or *B. jararacussu* venom (Jssu = 50 µg) pre-incubated with SAB or anti-PLA_2_ at the same rates used for the isolated fractions. After the incubation period, 50 µL of the mixtures were injected into the gastrocnemius muscle intramuscularly (i.m.) in groups of five mice. Three hours after injection, animals were bled from the orbital plexus, and the blood was centrifuged (2000× *g*, for 5 min) for further quantification of the serum CK levels, as described above.

### 5.9. Statistical Analysis

The statistical comparisons between the pairs were performed using the Student’s *t*-test. The statistical differences of more than two experimental groups were determined using a one-way analysis of variance (ANOVA), followed by the Tukey post-test, assuming a significance of *p* ≤ 0.05. The results were expressed as the mean ± standard deviation (SD). All data analysis was performed using GraphPad PRISM 8 (GraphPad Software, Inc.; La Jolla, CA, USA).

## Figures and Tables

**Table 1:** Values of main parameters of the coagulation via tromboelastometry.

	Human Plasma			Rat Plasma			Chicken Plasma		
	CT (s)	CFT (s)	MCF (mm)	CT (s)	CFT (s)	MCF (mm)	CT (s)	CFT (s)	MCF (mm)
Control (−)	857 ± 1.8	1349 ± 2.6	24.7 ± 5.3	609.6 ± 2.1	891 ± 2.5	26.1 ± 1.8	unclotted *	ND	ND
Control (+)	193.3 ± 1.4	861 ± 4.5	23.5 ± 1.3	134 ± 2.9	312.6 ± 3.1	22.3 ± 0.3	371 ± 1.5	431 ± 2.5	45.7 ± 0.5
FA1	260.3 ± 6.4	3143.3 ± 4	19.7 ± 0.9	273.6 ± 3.1	3061 ± 3.2	19.1 ± 0.5	458 ± 1.4	963 ± 3.9	38.3 ± 1.5
FA3	unclotted *	ND	19.7 ± 0.9	2248.5 ± 5.1	ND	11 ± 0.6	unclotted *	ND	ND
FA4	1549 ± 16.6	ND	17 ± 0.8	311 ± 3.4	ND	18.3 ± 0.4	597 ± 1.8	1349 ± 2.6	22.7 ± 5.3

* Unclotted until the end of experiment (1 h or 3600 s). ND—Not determined.

**Table 2:** Summary of the structural and functional features of the PLA_2_s isolated from *B. atrox* venom.

Fraction	pI	Residue at Position 49	Catalytic	Myotoxic	Edematogenic	Nociceptive	Anticoagulant	Antivenom Reactivity
FA1	8.4	K						
FA3	8.2	D						
FA4	5.1	D						

Color legend: Orange—represents high; yellow—intermediate, and blue—very low activity or reactivity with the commercial antivenom.

## Data Availability

The raw data relating to the results presented in this study are under the custody of the corresponding authors, and could be provided upon a plausible request.

## Supplementary Materials

The following supporting information can be downloaded at: https://www.mdpi.com/article/10.3390/toxins14080543/s1, Figure S1: reactivity of chromatographic fractions from *B. atrox* venom with the commercial *Bothrops* antivenom, in comparison with a species-specific antiserum.
