# Supplementary material for: Diversity of Phospholipases A2 from Bothrops atrox Snake Venom: Adaptive Advantages for Snakes Compromising Treatments for Snakebite Patients
Source: Toxins (Basel). 2022 Aug 8;14(8):543. doi: 10.3390/toxins14080543 (PMC9414272; doi:10.3390/toxins14080543)
Supplement: Supplementary file 1 [file toxins-14-00543-s001.zip › toxins-1839622-supplementary.pptx]

## Slide 1
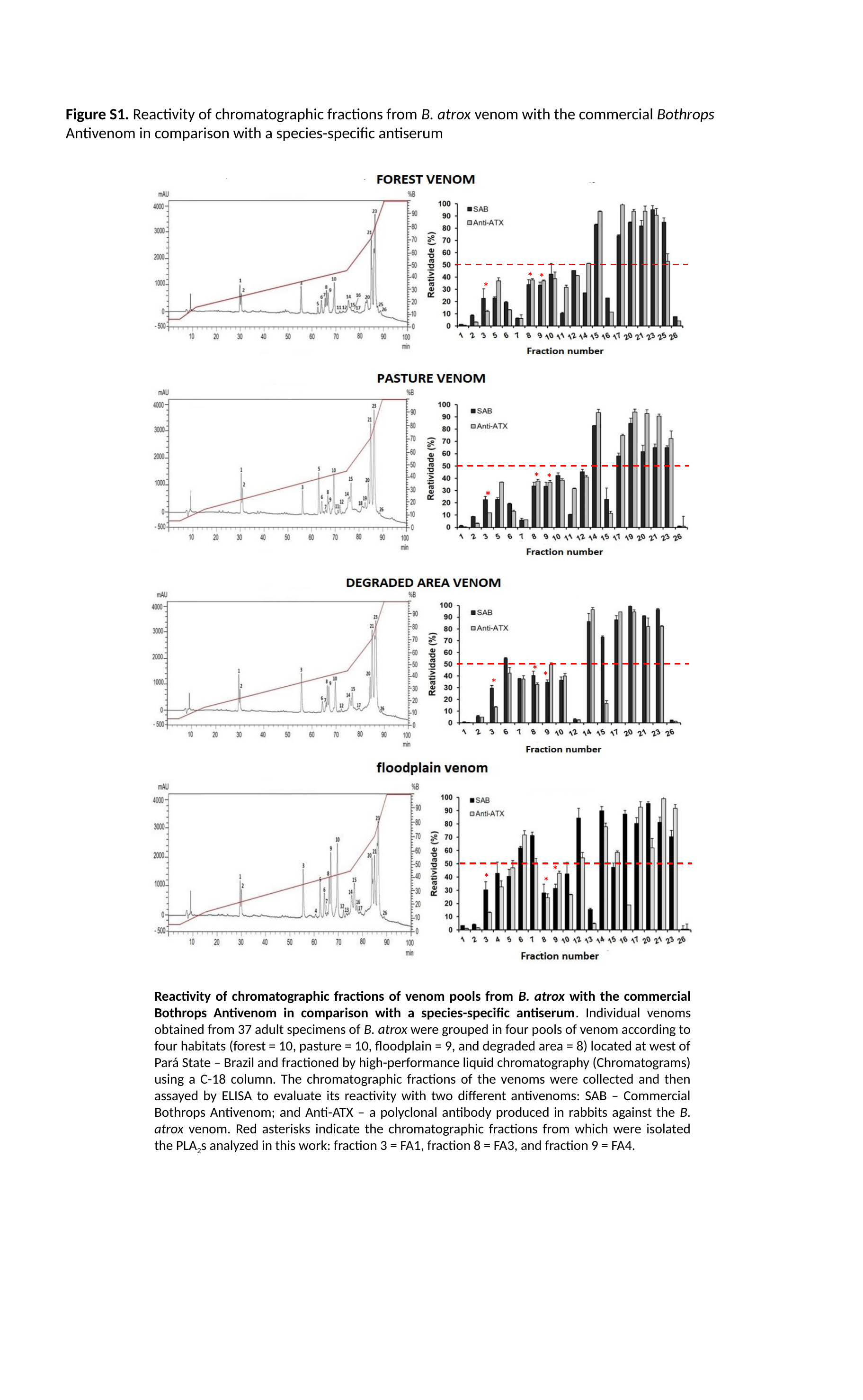

Figure S1. Reactivity of chromatographic fractions from B. atrox venom with the commercial Bothrops Antivenom in comparison with a species-specific antiserum
*
*
*
*
*
*
*
*
*
*
*
*
Reactivity of chromatographic fractions of venom pools from B. atrox with the commercial Bothrops Antivenom in comparison with a species-specific antiserum. Individual venoms obtained from 37 adult specimens of B. atrox were grouped in four pools of venom according to four habitats (forest = 10, pasture = 10, floodplain = 9, and degraded area = 8) located at west of Pará State – Brazil and fractioned by high-performance liquid chromatography (Chromatograms) using a C-18 column. The chromatographic fractions of the venoms were collected and then assayed by ELISA to evaluate its reactivity with two different antivenoms: SAB – Commercial Bothrops Antivenom; and Anti-ATX – a polyclonal antibody produced in rabbits against the B. atrox venom. Red asterisks indicate the chromatographic fractions from which were isolated the PLA2s analyzed in this work: fraction 3 = FA1, fraction 8 = FA3, and fraction 9 = FA4.
